# The dual amylin and calcitonin receptor agonist KBP-089 and the GLP-1 receptor agonist liraglutide act complimentarily on body weight reduction and metabolic profile

**DOI:** 10.1186/s12902-020-00678-2

**Published:** 2021-01-07

**Authors:** Anna Thorsø Larsen, Sofie Gydesen, Nina Sonne, Morten Asser Karsdal, Kim Henriksen

**Affiliations:** Nordic Bioscience Biomarkers and Research, Department of Endocrinology, Herlev Hovedgade 207, 2730 Herlev, Denmark

**Keywords:** DACRA, GLP-1, Obesity, Glucose tolerance

## Abstract

**Background:**

Weight loss therapy is becoming more and more important, and two classes of molecules, namely amylin receptor and GLP-1 receptor agonists, have shown promise in this regard. Interestingly, these molecules have several overlapping pharmacological effects, such as suppression of gastric emptying, reduction of glucagon secretion and weight loss in common; however, they also have distinct effects on prandial insulin secretion. Hence, a combination of these two mechanisms is of significant interest.

**Methods:**

In this study, we investigated the add-on potential of the dual amylin and calcitonin receptor agonist (DACRA) KBP-089 in combination with the GLP-1 receptor agonist liraglutide as obesity treatment in high-fat diet (HFD) fed rats.

**Results:**

Increasing doses of KBP-089 and liraglutide alone and in combination were studied with respect to their effects on body weight, food intake and glucose metabolism during a 9-week intervention study conducted in HFD rats. Further, the gastric emptying rate during an oral glucose tolerance was assessed. Treatment with KBP-089 and liraglutide dose-dependently lowered body weight 15% (at 2.5 μg/kg/day) and 7% (at 400 μg/kg/day) in HFD rats, respectively, while the combination resulted in a 21% body weight reduction, which was mirrored by reduction in fat depot sizes. Gastric emptying and glucose metabolism were improved, primarily by KBP-089, although liraglutide led to a reduction in fasting plasma glucagon.

**Conclusion:**

DACRAs complement GLP-1 on food intake, body weight, and glucose tolerance indicating the potential for an add-on therapy.

**Supplementary Information:**

The online version contains supplementary material available at 10.1186/s12902-020-00678-2.

## Background

Obesity is an increasing health problem due to modern lifestyle and excessive caloric intake. Multiple complications such as insulin resistance, type 2 diabetes, cardiovascular disease, cancer and non-alcoholic fatty liver disease, among others, are frequently associated with obesity [11, 15, 22]. A sustained weight loss is key in treatment of obesity; however, treatments beside lifestyle intervention are still few. Bariatric surgery is effective, but is only used in severe obesity due to risk of surgical complications [33], therefore alternative therapies with improved efficacy and low risk of side effects are of great interest. Furthermore, a significant weight loss is important in treatment of non-alcoholic fatty liver disease (NAFLD), non-alcoholic steatohepatitis (NASH), and other obesity related morbidities [7, 32].

Multiple GLP-1 agonists are approved for treatment of type 2 diabetes and recently high dose liraglutide was also approved for treatment of obesity, as it promotes sustained weight loss via effects on satiety and appetite [5, 39]. Additionally, liraglutide improves postprandial blood glucose concentration, although still with limitations in terms of tolerability challenges, in particular nausea [3, 8, 23, 27]. There is an increasing focus on combining GLP-1 receptor agonists with additional therapy to obtain greater therapeutic efficacy. In relation to combination therapy, amylin receptor agonism has raised significant interest as a possible candidate, since it has the potential for significant weight reduction and improved glucose control in both preclinical and clinical studies [2, 30, 38]. Additionally, pramlintide has been approved as adjunct to insulin therapy for treatment of type 1 and type 2 diabetes due to its ability to regulate post-prandial glucose levels, reduce body weight, and HbA1c [34, 37, 42]. Dual Amylin and Calcitonin Receptor Agonists (DACRAs) are specifically developed for their ability to activate both the amylin receptor and the calcitonin receptor, as well as their ability to induce typical amylin-induced responses, but with markedly superior potency [1, 12, 17, 30]. Importantly, GLP-1 and amylin analogues have several overlapping pharmacological effects including marked reductions in food intake, delay of gastric emptying and inhibition of glucagon secretion, although they act through different sites and mechanisms of action [36].

Previously, combinations of sub-optimal doses of the DACRA KBP-089 and the GLP-1 agonist liraglutide were shown to act complementarily on body weight, food intake and glucose tolerance [13, 14], indicating the add-on potential of KBP-089 to liraglutide in obesity treatment. As only sub-optimal doses of KBP-089 and liraglutide have been examined chronically, we here evaluate combination-effects of doses that elicits full response separately [13, 14]. In this study, we investigated the weight reducing potential of KBP-089 as monotherapy and in combination with the GLP-1 analogue liraglutide in obese high-fat diet fed (HFD) rats.

## Methods

### Peptide therapy

Synthetic KBP-089 (American Peptide Company, CA, USA) and liraglutide (SynPeptide, Shanghai, China) were dissolved in saline for subcutaneous delivery. The doses chosen for KBP-089 are based on [13, 14] and previous studies with liraglutide [13, 25].

### Animal experiments

All animal procedures were performed in accordance with the Animal Welfare Division of the Danish Ministry of Justice under license #2016-15-0201-00910. 108 male Sprague Dawley (SD) rats (Envigo, Horst, The Netherlands) were obtained at 6 weeks of age and housed as described previously [13, 14]. Obesity was induced by high fat diet feeding for 10 weeks as described by [13, 14].

The rats were allocated into treatment groups according to body weight (*n* = 8–10 rats/treatment group – 8 rats in monotherapy groups and 10 rats in combination therapy groups). The rats received doses of KBP-089 (KBP) (0.625, 1.25 and 2.5 μg/kg sc), liraglutide (L) (200 and 400 μg/kg sc) and the combinations (KBP 0.625 + L 200 μg/kg, KBP 0.625 + L 400 μg/kg, KBP 1.25 + L 200 μg/kg, KBP 1.25 + L 400 μg/kg and KBP 2.5 + L 200 μg/kg, KBP 2.5 + L 400 μg/kg) and vehicle (saline) for 9 weeks. Body weight and food intake were monitored daily in the initial three weeks, then once weekly. Following 4 and 8 weeks of treatment, oral glucose tolerance tests (OGTT) were performed. To assess the treatment effect on gastric emptying, rats received acetaminophen (40 mg/kg) p.o. gavage (4 mL/kg) together with the glucose bolus during OGTT and the appearance of acetaminophen in plasma was measured after 30 min. At study end the rats were euthanized, and epididymal, perirenal and subcutaneous inguinal fat depots were surgically removed and weighed.

### Glucose tolerance tests

OGTTs were performed at 4 and 8 weeks of treatment in rats fasted for 12 h. Rats were pre-dosed with either vehicle or drug at t = − 30 and the OGTTs were performed as described previously [9, 12–14].

### Biochemical analysis

Plasma samples for assessment of glucose, insulin and acetaminophen were collected and analysed as described by [13, 14, 17].

### Statistical analysis

The endpoints were: change in bodyweight, food intake, glucose tolerance and insulin levels.

All data are presented as mean ± standard error of the mean (SEM). The statistical analysis of group differences were assessed using one-way ANOVA followed by Tukey’s post-hoc test for multiple comparison. Statistical analyses of non-parametric data were conducted using Kruskal Wallis test followed by Dunn’s post-hoc test for multiple comparison. Normality of data distribution was determined by D’Agostino and Pearson test normality test. All analyses were performed using GraphPad Prism software (GraphPad Prism, San Diego, CA, USA). A value of *p* < 0.05 was considered statistically significant.

## Results

### KBP-089 acts complementary with GLP-1 on food intake and body weight loss

**Fig. 1:**
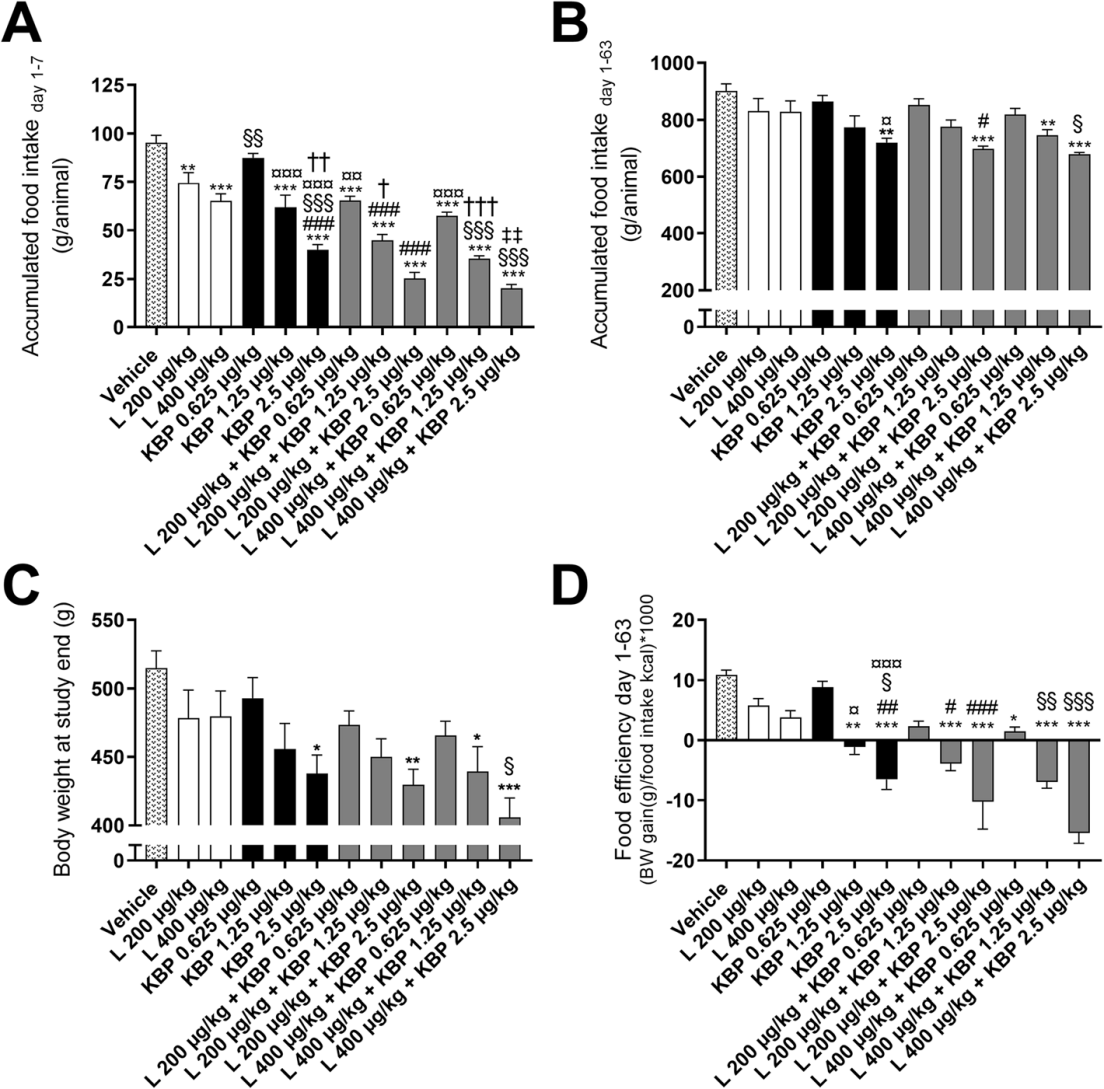
Accumulated food intake for the initial 7 days (**a**) and for the entire duration of the study (**b**). Body weights at study end (**c**). Calculated food efficiency (**d**). *N* = 8–10 rats per group. Statistical analysis between groups were performed as a one-way ANOVA followed by Tukey’s post-hoc test with the following annotations: *P < 0.05, **P < 0.01, ***P < 0.001 vs. vehicle, #P < 0.05, ##P < 0.01 vs. liraglutide (200 μg/kg), §P < 0.05, §§P < 0.01 vs. liraglutide (400 μg/kg), ^¤^ P < 0.05, ^¤¤^ P < 0.01, ^¤¤¤^ P < 0.001 vs. KBP-089 (0.625 μg/kg), † P < 0.05, †† P < 0.01, ††† P < 0.001 vs. KBP-089 (1.25 μg/kg) and ‡‡*P* < 0.01 vs. KBP-089 (2.5 μg/kg). All data are means ± SEM

To assess whether KBP-089 acts complimentary with GLP-1. KBP-089 and the GLP-1 analogue liraglutide were administered alone or in combination for 9 weeks in HFD rats. 9 weeks of treatment with KBP-089 (0.625, 1.25 and 2.5 μg/kg) resulted in a dose dependent weight loss (Fig. 1c and supplementary Fig. 1A). Chronic treatment with high concentrations of liraglutide (200 and 400 μg/kg) and KBP-089 (2.5 μg/kg) resulted in a 7 and 15% vehicle-corrected body weight loss, respectively, while the combinations (L 200 μg/kg + KBP 2.5 μg/kg and L 400 μg/kg + KBP 2.5 μg/kg) resulted in a 17 and 21% weight reduction, respectively (Fig. 1c and supplementary Fig. 1A). All treatments significantly reduced food intake in the initial phase of the study (Fig. 1a and supplementary Fig. 1B-C), while only high dose KBP-089 and combination therapy reduced food intake during the entire study (Fig. 1b). Based on food intake and body weight change, food efficiency was calculated. Treatment with the two highest KBP-089 doses (1.25 and 2.5 μg/kg) as well as their combinations with liraglutide resulted in a significant reduction in food efficiency compared with vehicle. Additionally, combination treatment was superior to treatment with liraglutide and showed a trend to towards superiority to KBP-089 alone (Fig. 1d).

### Treatment with KBP-089 and liraglutide reduces overall adiposity in high fat diet rats

**Fig. 2:**
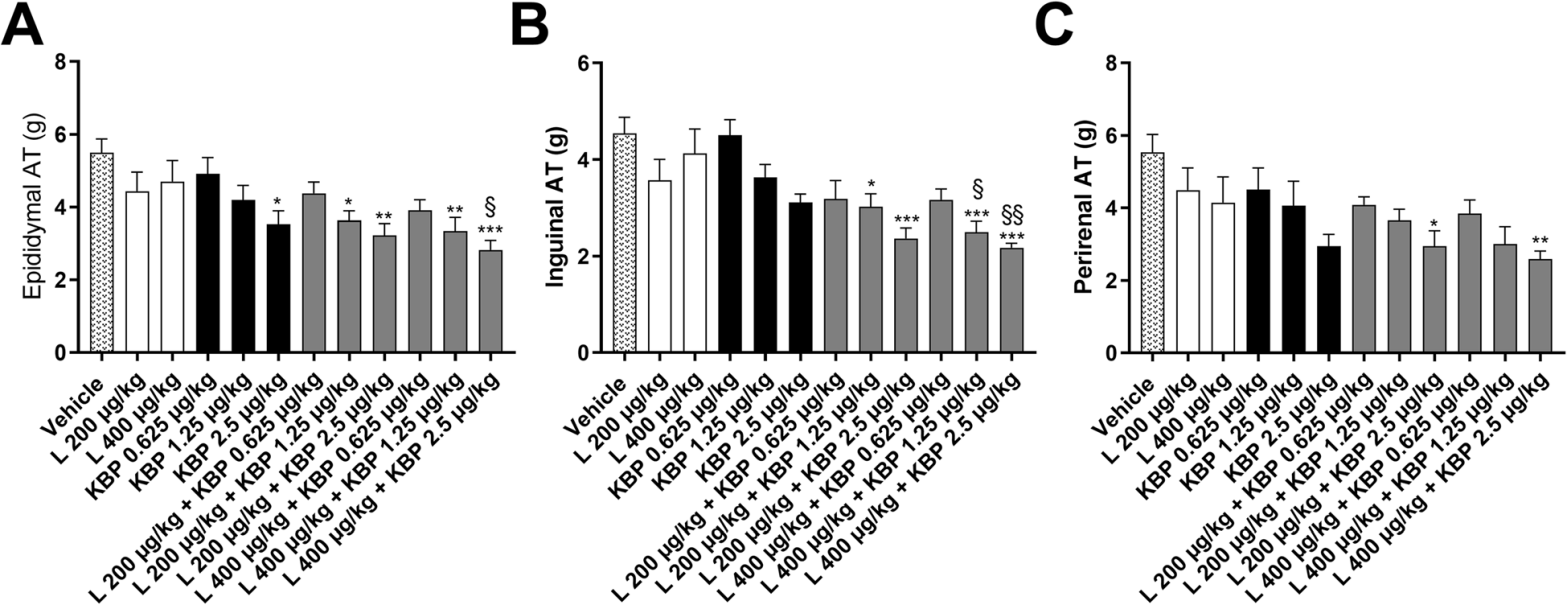
Weights of epididymal (**a**), inguinal (**b**) and perirenal (**c**) white adipose tissue (AT) at study end (*n* = 8–10 rats per group). Statistical analysis between groups (**a** and **b**) were performed as a one-way ANONA followed by Tukey’s post-hoc test and as Kruskal Wallis test followed by Dunn’s post-hoc test with the following annotations: *P < 0.05, **P < 0.01, ***P < 0.001 vs. vehicle, §P < 0.05, §§P < 0.01 vs. liraglutide (400 μg/kg). All data are means ± SEM

At study end adipose tissues were isolated and weighed. In conjugation with the significant reduction in body weight, the weight of epididymal white adipose tissues was significantly reduced after treatment with 2.5 μg/kg of KBP-089 and combinations of KBP-089 and liraglutide, whereas only the combination therapy significantly reduced the weights of inguinal and perirenal adipose tissue (Fig. 2a-c).

### Treatment with KBP-089 and liraglutide improves oral glucose tolerance with reduced insulin levels

**Fig. 3:**
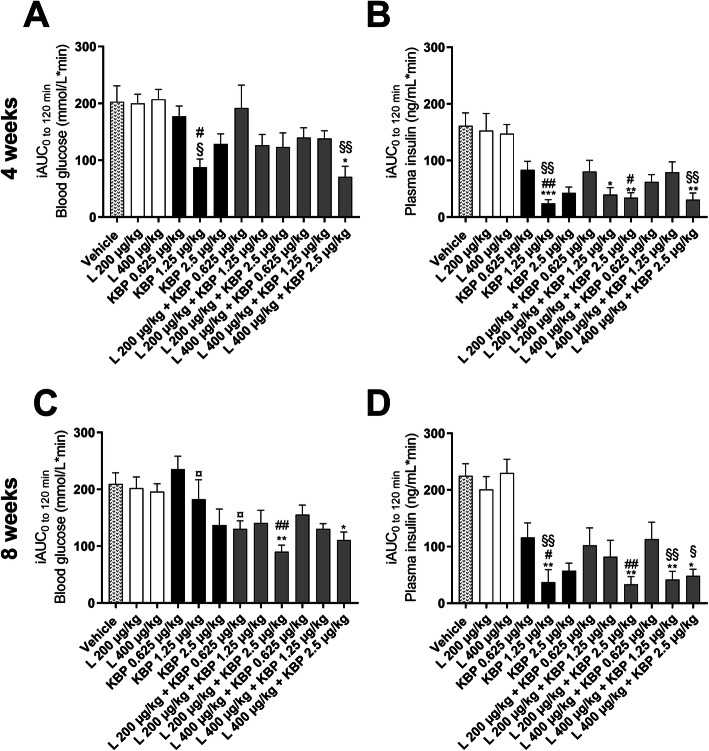
Oral glucose tolerance test (OGTT) after 4 and 8 weeks of treatment. The incremental area under the curve (iAUC) shown for blood glucose (**a** and **c**) and plasma insulin (**b** and **d**) during OGTT after 4 and 8 weeks, respectively. *n* = 8–10 rats per group. Statistical analysis between groups were performed as a one-way ANONA followed by Tukey’s post-hoc test (**c**) and as Kruskal Wallis test followed by Dunn’s post-hoc test (**a**, **b**, **d**) with the following annotations: *P < 0.05, **P < 0.01, ***P < 0.001 vs. vehicle, #*P* < 0.05, ##*P* < 0.01 vs. liraglutide (200 μg/kg), §*P* < 0.05, §§*P* < 0.01 vs. liraglutide (400 μg/kg), ^¤^ *P* < 0.05 vs. KBP-089 (0.625 μg/kg). All data are means ± SEM

OGTTs were performed after 4 and 8 weeks of treatment. After 4 weeks of treatment, KBP-089 (1.25 μg/kg) and the combination of high KBP-089 (2.5 μg/kg) and liraglutide (400 μg/kg) had decreased blood glucose levels, though only the combination significantly (Fig. 3a and supplementary Fig. 2A). During the OGTT after 8 weeks of treatment (Fig. 3c and supplementary Fig. 2C), the two combination groups (L 200 μg/kg + KBP 2.5 μg/kg and L 400 μg/kg + KBP 2.5 μg/kg) were able to significantly improve oral glucose tolerance considering the iAUC (Fig. 3c). After both short and long-term treatment (4 and 8 weeks) insulin levels were reduced in rats treated with KBP-089 (1.25 and 2.5 μg/kg) while unchanged in rats treated with liraglutide (200 and 400 μg/kg) compared to vehicle, resulting in significantly different iAUC values in KBP-089 and liraglutide treated rats (Fig. 3b,d). All combinations of the two treatments resulted in insulin levels in the same range as the KBP-089 treated rats, being significantly lower compared to vehicle. In addition, the combination of combination groups receiving the highest dose of KBP-089 (L 200 μg/kg + KBP 2.5 μg/kg and L 400 μg/kg + KBP 2.5 μg/kg) resulted in significantly lower insulin levels compared to groups treated with liraglutide alone (Fig. 3b,d and supplementary Fig. 2B,D).

### High dose Liraglutide, KBP-089 and the combination reduced fasting plasma glucagon levels

**Fig. 4:**
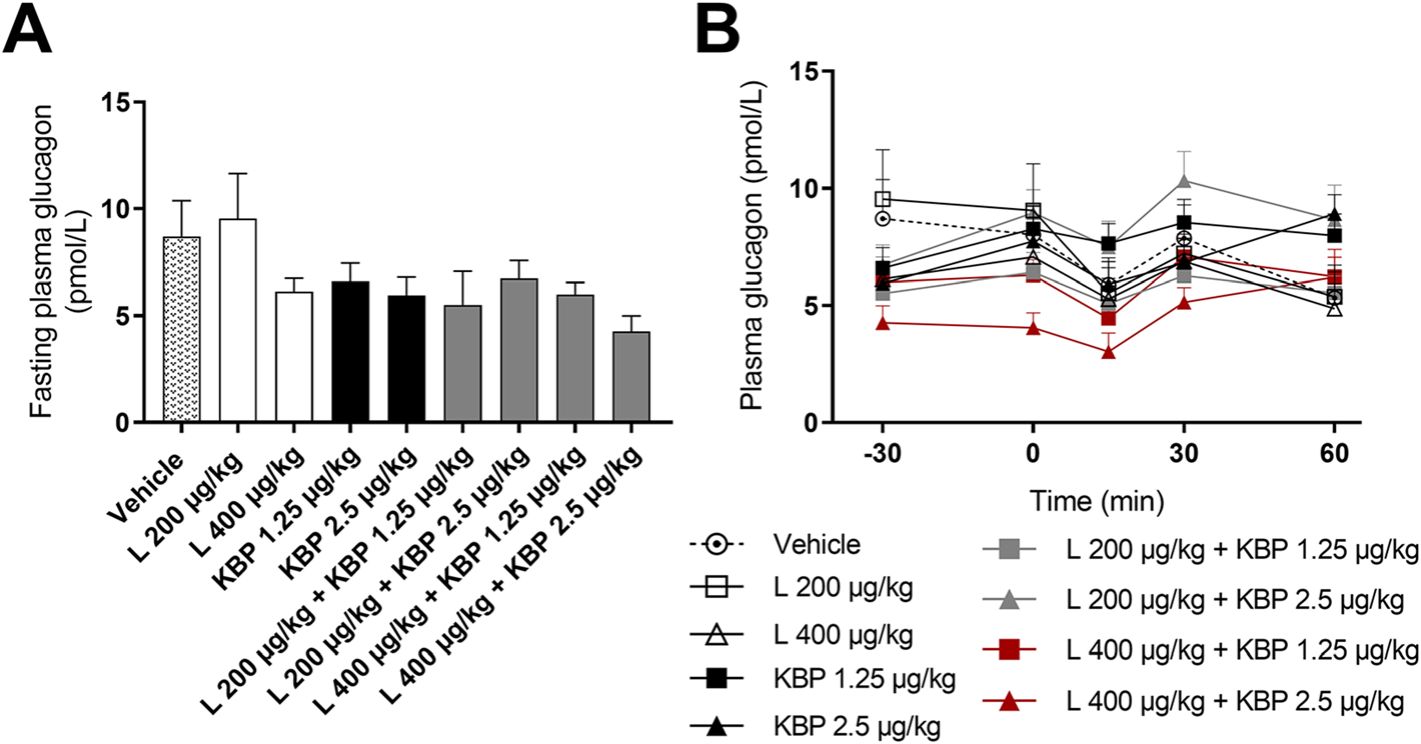
Fasting plasma glucagon levels (**a**) and plasma glucagon levels during OGTT after 8 weeks of treatment (**b**). *n* = 8–10 rats per group. Statistical analysis between groups were performed as a one-way ANONA followed by Tukey’s post-hoc test. All data are means ± SEM

Fasting plasma glucagon levels and glucagon levels during OGTT were assessed after 8 weeks of treatment. High dose liraglutide (L 400 μg/kg), KBP-089 (1.25 and 2.5 μg/kg) and the combination of slightly reduced fasted plasma glucagon levels compared to vehicle (Fig. 4a). Plasma glucagon levels during OGTT did not differ significantly between treatment (Fig. 4b).

### KBP-089 in combination with liraglutide delay gastric emptying rate

**Fig. 5:**
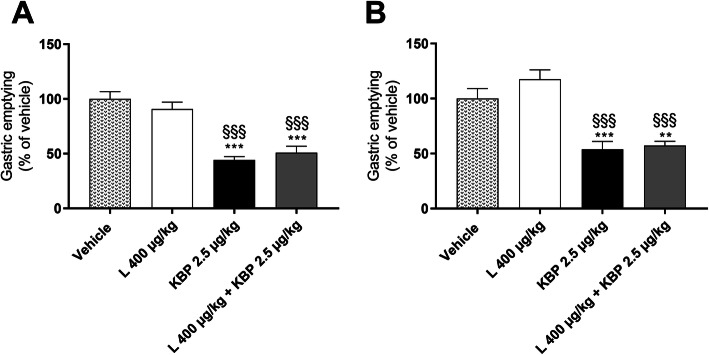
Gastric emptying after 4 (**a**) and 8 (**b**) weeks of treatment. Gastric emptying was estimated by the appearance of acetaminophen in plasma 30 min post dosing and calculated as % change relative vehicle. Statistical analysis between groups were performed as a one-way ANONA followed by Tukey’s post-hoc test with the following annotations: **P < 0.01, ***P < 0.001 vs. vehicle and §§§*P* < 0.001 vs. liraglutide (400 μg/kg). All data are means ± SEM

Gastric emptying was assessed in the treatment groups receiving the highest doses of KBP-089, high dose liraglutide and the combination of the two. The rate of gastric emptying during OGTT was assessed after 4 and 8 weeks of treatment (Fig. 5a,b). After both 4 and 8 weeks of treatment administration of KBP-089 (2.5 μg/kg) resulted in a significant reduction of gastric emptying rate 30 min after acetaminophen administration, while liraglutide (L 400 μg/kg) had no pronounced effect on gastric emptying (Fig. 5a,b). After 4 weeks of treatment liraglutide reduced gastric emptying by approximately 10% compared to vehicle (Fig. 5a), while liraglutide increased gastric emptying by approximately 18% compared to vehicle after 8 weeks of treatment (Fig. 5b). Additionally, the combination of high dose KBP-089 and liraglutide (L 400 μg/kg + KBP 2.5 μg/kg) significantly delayed gastric emptying, but equally to KBP-089 treatment alone. This effect on gastric emptying rate was unchanged from 4 (Fig. 5a) to 8 (Fig. 5b) weeks of treatment.

## Discussion

As there is a continuous need for increased potency on weight loss, we investigated the pharmacological potential of combination therapy using the highly potent DACRA KBP-089 and the GLP-1 analog liraglutide for obesity.

In this study, we found a significant effect on appetite suppression and body weight loss when combining the two peptides over a period of nine weeks, an effect superior to either monotherapy alone. Importantly, this also manifested in reduction in food efficiency and overall adiposity. Generally, KBP-089 was superior to liraglutide therapy, and the effects on body weight and food intake dose dependently followed KBP-089 concentrations when combing the two therapies. This suggests that KBP-089 is responsible for the majority of the efficacy of the combination therapy in this study. These findings correspond well with earlier observations using lower doses of the two peptides [13], and demonstrate an additive effect. Previous studies of KBP-089 using pair-feeding have demonstrated a weight loss beyond what is obtained through the reduction of appetite, and have clearly indicated that this effect likely entails increased energy expenditure, or at least a maintenance of energy expenditure, despite the reduction of food intake, a parameter known to reduce energy expenditure [12, 17, 29, 43].

In terms of glucoregulatory actions both amylin receptor agonism [31, 34, 37] and GLP-1R agonism [24, 39, 41] have shown potential. However, the glucose-lowering effect of GLP-1 receptor agonists involves increased post-prandial insulin secretion [26, 40]. During OGTT, both short and long-term treatment with KBP-089 improved glucose tolerance in accordance with previous studies performed with KBP-089 [13, 14]. Interestingly, the effect on blood glucose during OGTT was especially pronounced in combination therapy groups, particularly after eight weeks of treatment, supporting that the peptides act though complimentary pathways, and possibly that the combination leads to increased durability of the glucoregulatory effects compared to stand-alone treatment, consistent with the study by [28]. Importantly, along with improved glucose clearance, significantly lower insulin levels during OGTT were observed in KBP-089 (1.25 and 2.5 μg/kg) and combination therapy groups, indicating improved insulin sensitivity. It is likely that the majority of these effects is explained by the massive weight loss; however, DACRAs are known to directly suppress insulin secretion in an IVGTT, as well as directly on the pancreatic islets [1, 17], confirming weight independent effects. Secondly, studies applying pair-fed and pair-weighed controls, as well as studies in ZDF rats, which are insensitive to amylin receptor mediated weight loss [6], have documented glucose regulatory capacities beyond what is observed with weight loss [12, 17].

This together with the significant weight loss suggest potential not only as anti-obesity therapy, but also in treatment of obesity related co-morbidities such as type 2 diabetes and NASH [11, 22, 32]. Surprisingly, liraglutide did not increase plasma insulin as expected for a GLP-1 receptor agonist. Other studies in obese rats found similar lack of liraglutide induced increase in plasma insulin during OGTT [13, 35], suggesting that the lack of effect observed here might be explained by the animal model that is non-diabetic. Plasma glucagon levels were assessed after 8 weeks of treatment. All treatments, except liraglutide (200 μg/kg), tended towards a lowering of fasting plasma glucagon levels compared to vehicle. Though, all treatment groups had nearly constant glucagon levels during OGTT and no significant differences between groups were observed. This suggests that the HFD rat model does not show inappropriate elevated glucagon levels as seen in diabetic conditions and might explain why there is no clear effect of the therapies post glucose challenge.

GLP-1 and amylin analogues are both known to delay gastric emptying [36], hence gastric emptying rates were assessed. In accordance with earlier studies using DACRAs [16, 17], KBP-089 (2.5 μg/kg) markedly reduced gastric emptying after both short- and long-term treatment. A similar effect was observed in the group receiving high-dose combination therapy. Perhaps surprisingly, liraglutide alone only had minor effect on gastric emptying, even trending towards increasing vehicle-corrected gastric emptying after 8 weeks of treatment. Several clinical studies have shown that chronic treatment with liraglutide delays gastric emptying [10, 19, 39]. However, in a pre-clinical setting the ability of liraglutide to reduce gastric emptying markedly diminished within 14 days of treatment, explaining the lack of effect observed here [21]. The inhibited gastric emptying can positively affect postprandial blood glucose levels by delaying entry of glucose into circulation, a central factor in diabetes treatment. From a mechanistic point-of-view, a series of studies have looked into co-administration of either amylin or the DACRA salmon calcitonin (SCT) in combination with incretin-based therapies [4, 12, 28]. These studies have highlighted that both amylin and GLP-1 activate receptors in the same areas of the hind brain, i.e. the dorsal-vagal-complex (DVC), which contains the area postrema and the nucleus tractus solitarius [28]. These studies showed a combined effect of SCT and liraglutide on c-fos activation in the DVC, consistent with a combined suppression of food intake and gastric emptying [12, 28]. Furthermore, earlier work indicated that this effect may entail a local upregulation of brain IL-6 in the hypothalamus, by both amylin and GLP-1 [20]. Hence, while the complete picture of how the combination works is still unclear, there is evidence supporting that it entails common signaling pathways.

Importantly, there are some limitations to the study presented here. The weight lowering and glucoregulatory actions of both the mono- and the combination therapies are limited by model, as the HFD rat model does not develop diabetes, but only modest insulin resistance due to obesity. Furthermore, despite previous studies in diabetic model systems showing suppression of hyperglucagonemia [18], we only detected trends towards suppression of glucagon levels, most likely due to the model system only representing a mild disease. This is also seen for the weight loss, where the differences are rather small in the combination therapy arms of the study, as these seem to have reached maximal weight loss, albeit the lack of a lean control group confounds this conclusion. All in all, further studies in a diabetic model would be of importance.

In conclusion, KBP-089 acts complementary with the GLP-1 analogue, liraglutide, on food consumption, weight loss and glucose tolerance, indicating the potential for an add-on therapy causing additional improvement in metabolic profile.

## Supplementary Information

**Additional file 1.**

**Additional file 2.**

## Data Availability

The datasets used and/or analyzed during the current study are available from the corresponding author on reasonable request.

## Abbreviations

ANOVA: Analysis of Variance
DACRA: Dual Amylin and Calcitonin Receptor Agonists
ELISA: Enzyme-linked immunosorbent assay
GLP-1: Glucagon-like peptide 1
HFD: High Fat Diet
(i)AUC: (incremental) Area Under the Curve
OGTT: Oral Glucose Tolerance Test
SEM: Standard error of the mean
