# Supplementary material for: The dual amylin and calcitonin receptor agonist KBP-089 and the GLP-1 receptor agonist liraglutide act complimentarily on body weight reduction and metabolic profile
Source: BMC Endocr Disord. 2021 Jan 7;21:10. doi: 10.1186/s12902-020-00678-2 (PMC7791885; doi:10.1186/s12902-020-00678-2)
Supplement: Supplementary file 1 — Additional file 1. [file 12902_2020_678_MOESM1_ESM.docx]

**Supplementary Figure 1**

**Supplementary figure 1.** Body weight (A) and food intake (B) during the study. Accumulated food intake for the initial 14 (C). N=8-10 rats per group. Statistical analysis between groups in C were performed as a one-way ANOVA followed by Tukey’s post-hoc test with the following annotations: *P < 0.05, **P < 0.01, ***P < 0.001 vs. vehicle, ###P < 0.001 vs. liraglutide (200 µg/kg), §§P < 0.01 vs. liraglutide (400 µg/kg),  ^¤¤^ P < 0.01, ^¤¤¤^ P < 0.001 vs. KBP-089 (0.625 µg/kg). All data are means ± SEM.
