# Supplementary material for: The dual amylin and calcitonin receptor agonist KBP-089 and the GLP-1 receptor agonist liraglutide act complimentarily on body weight reduction and metabolic profile
Source: BMC Endocr Disord. 2021 Jan 7;21:10. doi: 10.1186/s12902-020-00678-2 (PMC7791885; doi:10.1186/s12902-020-00678-2)
Supplement: Supplementary file 2 — Additional file 2. [file 12902_2020_678_MOESM2_ESM.docx]

**Supplementary Figure 2**

**Supplementary figure 2.** Blood glucose (A and C) and plasma insulin (B and D) during oral glucose tolerance test (OGTT) after 4 and 8 weeks, respectively. n=8-10 rats per group. All data are means ± SEM.
